# Clinical Reports—New York College of Veterinary Surgeons

**Published:** 1898-05

**Authors:** 


### CLINICAL REPORTS—NEW YORK COLLEGE OF VETERINARY SURGEONS.

*Fracture of Ilium.* Bay horse, 15.3, working in furniture-van, picked up a nail, and while the driver was trying to pull it out, the horse fell over the pole, turning completely, and fractured the shaft of the ilium. Horse brought to hospital, condemned, and destroyed.

*Successful Operation on a Schirrous Cord, with Fistula.* Black gelding, eight years old. A rod composed of cotton, gum-arabic and bichloride of mercury, made about as thick as the lumen of the fistulous tube and about eight inches long, was forced into it. Considerable swelling of the scrotum followed, which gradually subsided, and on the fifth day the plug came away the length of the rod.

The horse was given internally one-half drachm of iodine crystals three times a day for five days; this was followed by one-half drachm doses of iodide of potash three times a day for a week.

Result: Obliteration of the fistula, tumor reduced two-thirds its size, and does not in the least interfere with the usefulness of the horse.

